# Radical Revelations: The Interplay of Nitrosative Stress, the Endocannabinoid System, and Treatment of Age-Related Disorders

**DOI:** 10.3390/ijms27062813

**Published:** 2026-03-20

**Authors:** Avery Davis, Isabella Y. Casmedes, Michael D. Burton

**Affiliations:** Neuroimmunology and Behavior Lab (NIB), Department of Neuroscience, School of Behavioral and Brain Science, Center for Advanced Pain Studies (CAPS), University of Texas at Dallas, Richardson, TX 75080, USA; avery.davis@utdallas.edu (A.D.); isabella.casmedes@utdallas.edu (I.Y.C.)

**Keywords:** endocannabinoid, reactive nitrogen species, redox environment, nitrosative stress, nitrergic signaling, Alzheimer’s, Parkinson’s

## Abstract

The crosstalk between the endocannabinoid system (ECS) and reactive nitrogen species (RNS) has emerged as an important area of investigation in recent years. Although many aspects of this interaction remain elusive, accumulating evidence demonstrates that the ECS plays a critical role in regulating RNS-mediated signaling under physiological conditions. This modulation can be either inhibitory or stimulatory, depending on the specific receptor subtype, cell type, and tissue location involved. While ECS-RNS interactions support normal cellular homeostasis, their dysregulation contributes to various disease states, particularly neurodegenerative disorders. Studies in both rodent models and human subjects show that ECS modulation can reduce anxiety, attenuate neuroinflammatory responses, and slow disease progression in neurodegenerative conditions. This review examines how cannabinoid-based interventions modulate nitrosative stress and neuroinflammation in Alzheimer’s disease (AD) and Parkinson’s disease (PD), highlighting their potential as targeted therapeutics that address multiple pathological mechanisms simultaneously and may offer advantages over conventional treatment approaches.

## 1. The Endocannabinoid System

### 1.1. Synthesis

The endocannabinoid system (ECS) is an endogenous neuromodulatory system critical to synaptic plasticity, central nervous system development, and cellular signaling [1]. The endocannabinoid system is composed of G protein-coupled receptors (GPCRs) such as CB1R and CB2R, endocannabinoids, and many enzymes such as fatty acid amide hydrolase (FAAH) which aid in the synthesis and degradation of endocannabinoids [2,3,4]. Cannabinoids are broadly split into endogenous cannabinoids (AEA and 2-AG), phytocannabinoidstetrahydrocannabinol THC) and cannabidiol (CBD), and synthetic cannabinoids (dronabinol and nabilone) which all vary in ECS activation and subsequent downstream signaling cascades [5,6]. Ubiquitous expression of cannabinoid receptors and their numerous signaling pathways involves this system in many biological functions such as hunger, memory, learning, metabolism, sleep and mood regulation [7,8].

**Figure 1:**
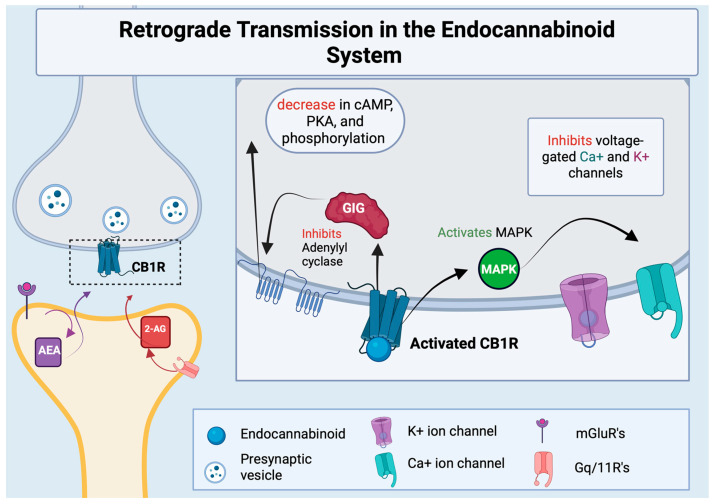
Retrograde transmission in the endocannabinoid system. Arachidonoyl ethanolamide (AEA) and 2-archidonoyl glycerol (2-AG) are synthesized at the post-synaptic neuron signaled by an activated receptor or voltage-gated channels respectively. Activation of the CB1 receptor on the presynaptic neuron inhibits adenylyl cyclase, the production of cyclic adenosine monophosphate (cAMP), protein kinase A (PKA), and overall phosphorylation. CB1R activation also activates mitogen-activated protein kinase (MAPK) which goes on to inhibit voltage-gated channels, preventing the firing of the cell. Created with BioRender.com.

Endocannabinoids are endogenous lipids that activate cannabinoid receptors, the most well studied of which are arachidonoyl ethanolamide (AEA) and 2-archidonoyl glycerol (2-AG) [1]. AEA is generated from a membrane phospholipid precursor enzyme known as N-acylphosphatidylethanolamine (NAPE) spurred by the enzyme NAPE-hydrolyzing phospholipase D (NAPE-PLD) [9,10]. 2-AG production is mainly activated by phospholipase C (PLC)-attenuated cleavage of membrane phosphatidylinositols, which activates a diacylglycerol (DAG) precursor [11]. Hydrolysis via DAG-lipase activity then initiates the formation of 2-AG [12]. Endocannabinoids are synthesized at the post-synaptic neuron and can easily pass through the phospholipid bilayer to activate CB1Rs on the presynaptic neuron, in a process known as retrograde transmission [13,14]. This retrograde signaling allows endocannabinoids to mediate both short-term and long-term plasticity for both excitatory and inhibitory synapses, as demonstrated in Figure 1.

### 1.2. ECS Receptors Differentiation and Function

Within the ECS, the most well-known and thoroughly studied receptors are CB1R, CB2R, and TRPV1 [15,16]. Each receptor is densely populated in specific tissues with CB1R found primarily in the central nervous system (CNS) and CB2R mainly located on immune cells [7]. While TRPV1 has been found throughout the central and peripheral nervous system, they are primarily located on peripheral sensory neurons [15,16]. CB1R and CB2R are unique GPCR’s in that they are able to recognize and bind with more than one endogenous ligand [17,18].

TRPV1 receptors, the least understood of the cannabinoid receptors, are non-selective cation ligand-gated channels highly permeable to Ca^2+^ [16]. Activation of the TRPV1 receptor causes an influx of calcium, resulting in muscle contractions, neurotransmitter release, and membrane excitability [19,20]. TRPV1 receptors, activated by noxious heat and certain vanilloids such as capsaicin, play a role in initiating thermal nociception [20,21]. Previous studies have utilized TRPV1 antagonists, namely capsazepine or AMG9810 to reduce chronic intractable pain due to cancer, migraines, AIDs, and chronic cough via its anti-inflammatory properties, demonstrating therapeutic potential for its usage in chronic pain disorders [16].

The location of these different cannabinoid receptors within the body are often indicative of their effects. CB1R activation can lead to changes in behavior, memory, movement, and cognition with high concentrations in the neocortex, hippocampus, basal ganglia, cerebellum, and brainstem [22,23]. Clinical trials found activation of CB1R alone showed promising results in ameliorating the psychological and behavioral symptoms of dementia such as reductions in nocturnal motor activity, aggression, anxiety-related motor activity (e.g., hand wringing) and restlessness [24,25]. The authors theorized these changes may be due to neurotransmitter modulation, reduced oxidative stress and neuroinflammation, and reduced tau hyperphosphorylation as well as reduced amyloid-related neurotoxicity [24,25]. Moreover, activation of CB1R in the CNS has been shown to inhibit murine glutamate release, decreasing excitatory signaling between neurons and decreasing anxious behaviors [2,26,27]. Studies where mice lacking CB1 receptors reliably show increased avoidance in tasks measuring anxious behaviors, including the Elevated Plus Maze (EPM), Open Field Test, and the Light-Dark Box Task [22]. These and other similar findings bolster the suggested role of endogenous CB1 receptors in anxiolytic functions. Additionally, CB1R deletion from GABAergeic interneurons specifically showed increased anxious behavior, whereas deletion from glutamatergic neurons were associated with a lower stress response [28].

The CB2 receptor, found primarily on immune cells, has therapeutic potential for diseases characterized by inflammatory dysregulation via modulation of immune activity and release of inflammatory mediators [29,30]. Importantly, inflammatory dysregulation is a hallmark of nitrosative and oxidative stress contributing to neurodegeneration, particularly in disorders like Alzheimer’s (AD) and Parkinson’s (PD). These associations lead to the possibility of new therapeutic developments involving the modulation of these receptors in the alleviation of cognitive impairments and maladaptive inflammation. However, further investigation into the cellular circuity of cannabinoid receptors and its effects on the inflammatory microenvironment is needed.

### 1.3. Mitochondria, RNS, and Nitrosative Stress

Mitochondrial dysfunction [31,32,33] is linked to a wide range of conditions, including neurodegenerative diseases like Parkinson’s and Alzheimer’s, metabolic disorders, and aging. Mitochondria are responsible for producing most of the cell’s energy in the form of ATP (adenosine triphosphate) through cellular respiration [34]. Mitochondria have a distinctive double-membrane structure [32], with the inner membrane being heavily folded into structures called cristae. These folds increase surface area for energy production, but they are also the source of mitochondrial nitric oxide synthase (mtNOS), a major driver in reactive nitrogen species (RNS) production. Mitochondrial mechanisms such as fission, fussion, and mitophagy are crucial for the regulation of their structure and functionality [33,35,36]. As an organism ages, structural features of mitochondria are altered and can result in physiological flaws such as decreases in mitochondrial dynamics and decreased functioning [36,37]. Such as example can be seen in the cristae structure of mitochondria, the location of oxidative phosphorylation [36]. Vue et al. found that mitochondrial morphology in aged rodent muscle cells display an overall smaller size and decrease connectivity of mitochondrial networks [36].

Reactive nitrogen species are nitric-oxide-derived compounds with unique cell signaling mechanisms that result in post-translational modifications and interactions with reactive oxygen species (ROS) [38]. However, increased levels of RNS can induce cellular damage, causing a decrease in cellular functioning and apoptosis [39]. Over time, it has become clear that RNS are not solely detrimental to the cellular environment but play a necessary role in retrograde cellular signaling. Regular levels of RNS are crucial for brain functioning—including synaptic plasticity—but can become detrimental if RNS levels are elevated [40,41]. This network is mediated by both endogenous and exogenous antioxidants (AO), molecules that exhibit a neuroprotective capacity via inhibition of lipid peroxidation which can induce cellular damage [42,43]. An imbalance of AO and RNS results in nitrosative stress, causing disastrous effects on cellular health—including alterations to protein structure, subsequent impairment of mitochondrial function, and disruption of cellular signaling [44,45]. This discord in the microenvironment is implicated in diseases such as Alzheimer’s, Parkinson’s, multiple sclerosis (MS), and several other neuroinflammatory disorders [46].

In the brain, neuronal mitochondria are the main source of free radicals, specifically in Complex I and III of the electron transport chain [47]. At physiological levels, mitochondrial ROS serve essential neuronal functions modulating synaptic plasticity, Ca^2+^ channels, and Ca^2+^/Calmodulin-dependent protein kinase II (CaMKII) to support long-term potentiation, the cellular mechanism underlying learning and memory [48]. ROS exert these effects by directly modifying mitochondrial Ca^2+^ transport proteins which subsequently alters mitochondrial Ca^2+^ uptake [49]. ROS exerts these effects by directly modifying mitochondrial Ca^2+^ transport proteins, thereby altering mitochondrial Ca^2+^ uptake, and by activating ryanodine receptors (RyRs) on the endoplasmic reticulum (ER) membrane to trigger Ca^2+^ release [49]. The ER and mitochondria are physically coupled at mitochondria endoplasmic reticulum contact sites (MERCs), where the close proximity creates high local Ca^2+^ concentrations that facilitate efficient transfer into the mitochondria matrix through the mitochondrial calcium uniporter (MCU) complex. Initially, increased mitochondrial Ca^2+^ uptake boosts oxidative phosphorylation, however, excessive Ca^2+^ triggers the opening of the mitochondrial permeability transition pore (mPTP), amplifying ROS release and initiating cell death pathways including apoptosis and autophagy [50].

Mitochondrial Ca^2+^ uptake promotes ROS generation, further triggering Ca^2+^ release from the ER, and enhancing mitochondrial uptake of Ca^2+^ at MERCs [50]. This positive feedback loop promotes further ROS generation and forms a cycle of damage. Excess ROS levels overload mitochondrial Ca^2+^ levels, triggering the opening of mPTP and leading to mitochondrial dysfunction and cell death [50].

Nitric oxide (NO) is a reactive nitrogen species produced by most cells in the conversion of l-arginine to citrulline and is unique in its ability to cross the phospholipid barrier and bind with the presynaptic neuron [51]. NO upregulation can be activated by three differing nitric oxide synthases (NOS): neuronal NOS (nNOS), endothelial NOS (eNOS), or inducible NOS (iNOS) [51]. Both nNOS and eNOS are enzymes that require calmodulin and calcium to become activated, whereas iNOS is induced in response to an inflammatory event such as neurodegenerative disease, tissue injury, hypoxia, and specific pro-inflammatory mediators like cytokines [52,53]. NOS in a balanced system are necessary for synaptic plasticity and long-term potentiation (LTP), a process critical to strengthening synaptic connections as well as memory formation. Following N-methyl-D-aspartate (NMDA) receptor activation by NOS, NO acts as a retrograde messenger to the presynaptic neuron via upregulation of the enzyme guanylate cyclase, resulting in an increase in cyclic guanosine monophosphate (cGMP) and subsequent enhanced release of glutamate [40]. This cascade results in sustained and amplified synaptic signaling [40].

N-omega-Nitro-L-arginine methyl ester (L-NAME), a non-selective NOS inhibitor, has been shown to decrease NO levels and negatively affect long-term potentiation [54]. In a study by Hopper and Garthwaite [55], rodents were injected with L-NAME and received continuous stimulation to an area of their hippocampus. Extracellular recordings of the field excitatory post-synaptic potentials (fEPSPs) were measured to investigate the effect of NOS on LTP. In healthy controls, the fEPSPs became elevated for the duration of the stimulus—which results in the LTP. However, rodents injected with L-NAME showed decreased hippocampal firing an hour after continuous stimulation—suggesting blockade of NOS by L-NAME obstructed LTP due to the failure to maintain synaptic response over time [55]. In the same study, administration of l-arginine—a precursor to NOS activation—reversed the effects of L-NAME on LTP, emphasizing the necessity of NOS activation for the formation of LTP.

Phosphorylation of nNOS by cAMP -dependent protein kinase [42], protein kinase C (PKC) [56], or CAMKII has been shown to decrease nNOS activity, and therefore decrease NO production [57]. cAMP, PKC, and CAMKII NO have been found to activate soluble guanylyl cyclase, a catalyst to the production of cGMP [56]. This cyclic guanosine monophosphate is a key messenger thought to indirectly activate protein kinase G (PKG), which significantly impacts protein phosphorylation [58,59].

Cannabinoid agonists can stimulate the production of cGMP and the translocation of guanylyl cyclase in neurons, influencing hyperpolarization-activated cyclic nucleotide gated channels, leading to changes in synaptic plasticity and long-term potentiation [27,60]. These shared signal transduction mechanisms imply a prospective functional relationship between the ECS and RNS systems as cAMP, PKC, and CAMKII all participate in ECS receptor activation and subsequent downstream signaling, although the exact interaction has yet to be fully elucidated [61].

NO can directly bond to reactants in a process known as nitrosylation, a process where NO-derived free radicals can form nitrate proteins and lipids effectively changing their functionality, and wreaking havoc upon the cellular environment [62,63]. The combination of NO and a superoxide anion form peroxynitrite (ONOO-) which is a highly reactive anion shown to impede cellular functioning, via lipid peroxidation [62,63]. ONOO-build-up has been proven to increase post-translational modification of DNA including protein nitration as well as perturb cellular functioning via lipid peroxidation and/or DNA degradation [64,65]. The addition of a nitrosonium ion (NO+) to a nucleophilic group (nitrosation) has similarly been proven to have a key role in cellular signaling while also negatively contributing to the accumulation of RNS [40]. In a study using s-nitrosation (nitrosation of a cysteine thiol) of connexin 43 within murine cardiac tissue, researchers found increased mitochondrial permeability—particularly for potassium ions—resulting in increased ROS formation and disequilibrium of the cellular redox system [66]. Nitration, the addition of a nitro group (-NO2) into amino acid debris, has proven to make functional alterations in proteins, such as heat shock protein 60 and 90, which reside within the mitochondria, and maintain protein homeostasis [67,68,69,70]. DNA and mitochondrial damage are linked to many age-related disorders, suggesting the blockage of excess nitration as a possible intervention.

### 1.4. Molecular Mechanisms of Mediation

The interaction between the ECS, the accumulation of RNS, and nitrosative stress, although yet to be fully understood, provides potential treatment avenues for NOS-related disorders. It has proven more difficult to examine the crosstalk of the ECS and RNS systems in healthy individuals, although some in vitro studies have suggested autocrine regulation to be one of the many regulatory functions of the interaction between the ECS and nNOS [61]. Moreover, CB1R and nNOS proteins are found in higher concentrations within the hypothalamus, suggesting functional interactions between these systems and hormone regulation [71].

**Figure 2:**
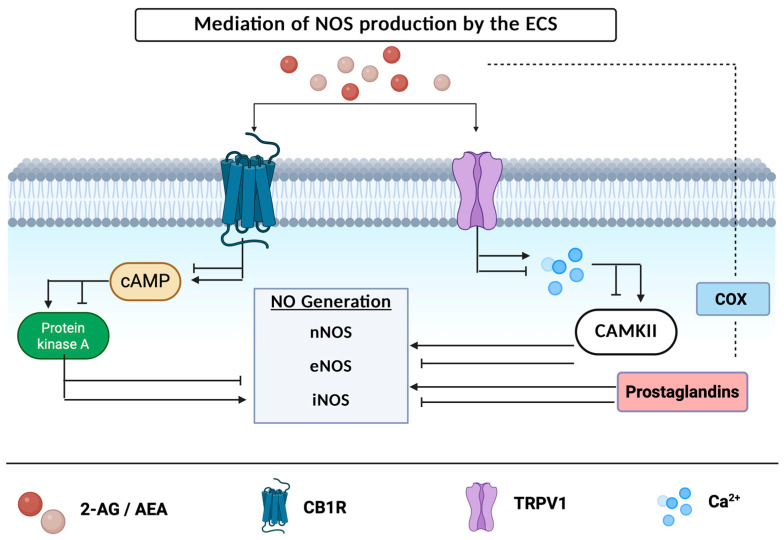
Mediation of NOS production by the ECS. Endocannabinoids AEA and 2-AG activate the CB1 or TRPV1 receptor are broken down by COX enzyme into prostaglandin metabolites. CB1R activation leads to either inhibition or stimulation of cAMP, PKA. Created in BioRender.com.

Activation of either CB1R or CB2R can impede or ameliorate the activity of enzymes responsible for the synthesis of NO [72,73]. Kim et al. found that NOS activity is enhanced within the cortex of CB1R knockout mice, indicating that RNS levels are mediated by activation of the CB1 receptor [23,74]. Cannabinoid receptor activation may either suppress or increase the levels of cAMP within the cellular environment, which in turn inhibits or activates the PKA-attenuated production of enzymes responsible for the production of NOS [75] (Figure 2). AEA activation of CB1R can inhibit the activation of voltage-gated calcium channels, preventing the production of action potentials [62]. In rodent models, activation of CB1R inhibits the release of glutamate, decreasing neuronal excitation [22]. In contrary, activation of the TRPV1 receptor results in an influx of sodium and calcium, increasing neuronal excitation [16] and demonstrating that ECS receptors have drastically differing effects. NOS production can be modulated by an increase or decrease in action potential firing with subsequent increases or decreases in calmodulin binding and the activity of CAMKII [72]. When CAMKII, an enzyme responsible for decreasing nNOS activity and NO production, is inhibited, there is an increase in NO levels along a simultaneous decrease in superoxide generation—leading to a profound nitrosative imbalance [76,77]. Furthermore, the recycling of endocannabinoids by the COX enzyme, which converts arachidonic acid into prostanoids, results in prostaglandin metabolites that have been shown to mediate NOS activity shown in Figure 2 [72].

A potential mechanism of neuroprotection is microglial CB2R activation which attenuates iNOS expression, shifting from a pro-inflammatory to an anti-inflammatory phenotype [78]. AM1241, a CB2 agonist, has been shown to attenuate microglial activation via reducing the expression of iNOS in rodent models, implicating the role of the ECS in the modulation of iNOS [79]. Furthermore, inhibition of FAAH via PF-3845 in a TBI mouse model resulted in suppressed iNOS expression alongside COX-2, whilst elevating AEA levels and thus reducing neurodegeneration [80]. Esposito et al. found that treating rat glioma cells with ACEA—a full CB1 agonist—prior to exposure of Abeta significantly reduced iNOS protein expression. Additionally, treating Abeta-stimulated cells with ACEA significantly reduced tau hyperphosphorylation in a concentration-dependent manner [81]. Studies utilizing WIN-55,212-2, a dual CB1/CB2 agonist, have found an overall increase in cell viability, while decreasing pro-inflammatory cytokines, COX-2 accumulation, and iNOS production [82]. These findings suggest that the ECS counteracts the iNOS inflammatory cascade at multiple levels.

The complex dynamics between the ECS and NOS are widely variable, and subsequent cellular effects are dependent on tissue type, ECS receptor expression, and NOS specificity. While more research is needed to parse out the interactions of the ECS and NOS in healthy individuals, current data shows promise for the development of therapeutics that work in combination to target these systems in neurodegenerative disease states.

ECS modulation of NOS activity extends to peripheral immune contexts. Gu et al. found that monoacylglycerol lipase (MAGL) accumulates in synovial tissue from patients with osteoarthritis (OA) alongside iNOS-positive M1-macrophages. Macrophages, an innate immune cell, is a key location of iNOS production [83]. Within the same study, mice in an OA model also displayed elevated MAGL and iNOS in synovial tissue [84]. Pharmacological inhibition of MAGL shifted macrophage polarization from the pro-inflammatory M1 phenotype, characterized by high levels of cytokines and iNOS, towards the anti-inflammatory M2 phenotype, characterized by increased arginase 1 expression. This transition was accompanied by reduced MAGL and iNOS expression, increased pain thresholds, and lower inflammation scores [84]. These findings suggest MAGL inhibition as a mechanism of iNOS expression in macrophages, providing an example of ECS-NOS crosstalk with direct implications for inflammatory disorders.

## 2. Therapeutic Potential of Cannabinoids in Aging and NOS-Related Disorders

### 2.1. Alzheimer’s Disease

Aso and colleagues [85] investigated the interaction between AD and the ECS utilizing knockout CB1R mice with the APP/PS1 genes—causing the animal to exhibit an early onset AD phenotype. They analyzed mice with a knockout of CB1R (−/−), heterozygous CB1R mice (−/+), and mice with overexpression of the CB1R gene (*Cnr1*). The absence of CB1R drastically reduced the survival rate of APP/PS1 mice, likely due to seizures caused by the imbalance of excitatory and inhibitory signaling [85]. Mice with reduced expression of CB1R (−/+) displayed accelerated memory decline between 3 and 6 months of age for mice with the AD phenotype [85,86]. A possible explanation for these findings is decreased levels of PSD-95 protein, a scaffolding protein within excitatory neurons, indicating synaptic dysfunction [87]. Overexpression of CB1R led to a more substantial decline in memory, demonstrating that the imbalance of CB1R causes deficits in memory retrieval and acquisition, similar to NOS accumulation.

Postmortem studies of brains taken from patients with AD have increased expression of CB2R’s on microglia within senile plagues [88,89]. However, CB1R expression is reduced in neurons distal to senile plaques [90,91]. FAAH, an endocannabinoid metabolizing enzyme, has also been found to be upregulated in senile plaques [92] and may contribute to the upregulation of anandamide metabolites. This cascade may be involved in increasing production of prostaglandins and pro-inflammatory cytokines in the AD brain.

**Figure 3:**
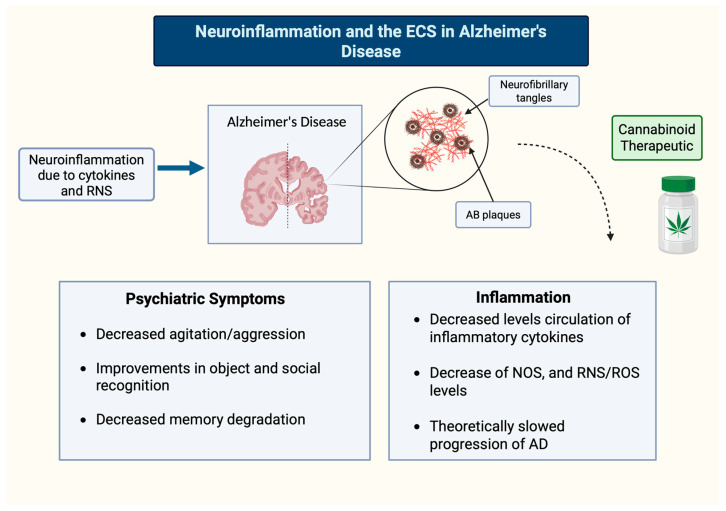
Neuroinflammation and the ECS in Alzheimer’s Disease.

The presence of FAAH in astrocytes proximal to senile plaques may participate in the reactive gliosis in regions with excess Aβ deposits [92,93]. These findings imply a compensatory mechanism in the AD brain to moderate levels of RNS via CB1R microglial expression. In rats with AD-type pathology, deposits of Aβ plaques and cognitive impairment were reversed by the non-selective CB1 and CB2 agonist WIN-55 212-22, and the CB2 selective agonist JWH-133 [92]. CB2R manipulation was only associated with activated microglia within or proximal to the plaques [92]. These findings imply a compensatory mechanism in the AD brain to moderate levels of RNS via CB receptor microglial expression. Benito and colleagues [92] hypothesize that the neuroprotective properties of cannabinoids are likely to include a downregulation of activity of transcription factors that lead to the production of inflammatory cytokines. The modulation of these inflammatory pathways could be used to treat AD pathology by targeting CB2R via its ability to halt or reverse the neuroinflammatory cycle (Figure 3).

### 2.2. Parkinson’s Disease and MS

NO can mediate neurotoxicity [94] and seems to play a role in the progression of neuroinflammatory disorders. Rodent models of Parkinson’s disease, a hypoactive motor disorder, treated with an NOS inhibitor or have a deletion of nNOS and iNOS genes, have significantly less MPP^+^-induced neurotoxicity [95,96]. Inhibition of NO production can therefore serve a protective role in decreasing DNA damage and cellular dysfunction. MS, another condition characterized by persistent neuroinflammation, is also impacted by NO production [61]. In the clinical population, high levels of NO are seen not only in MS lesions, but also in patients’ urine, cerebrospinal fluid, and blood [97,98]. Additional research has proposed that NO production is also related to demyelination in the central nervous system commonly seen in MS [99,100].

A wealth of literature is lacking on the use of cannabinoids for PD related motor symptoms, but several studies have found that high urine concentrations of 11-OH-THC, the active metabolite of THC, were associated with improved tremors, rigidity, and bradykinesia [101,102,103]. In the 6-OHDA rat model, tetrahydrocannabivarin (THCV) was demonstrated to have symptom-reducing effects by reducing motor inhibition, improving akinesia/bradykinesia, and neuroprotection of nigral dopaminergic neurons [104,105].

THCV, another compound derived from cannabis, has proven to be an interesting method of treatment for motor symptoms related to Parkinson’s, due to its antagonism of CB1R but activation of CB2R [106,107]. Parkinsonian rats that received acute injections of THCV displayed motor inhibition similar to the effect of rimonabant, a CB1R antagonist, through alterations in glutamatergic transmission in the basal ganglia [104]. Long-term administration of this compound also decreased microglia activation and helped conserve nigrostriatal dopamine neurons [108,109]. Espadas et al. found that THCV administration delayed initial dyskinesia and reduced intensity of existing dyskinesia in a mouse model of Parkinson’s disease [105].

Clinical trials for the use of cannabinoids for MS have provided mixed results. Smaller studies found that Dronabinol was more efficacious than placebo at decreasing spasticity [110,111,112,113]. However, other studies [112] found no differences between cannabinoids and placebo treatment on spasticity relief. Mixed results have been hypothesized to be in part due to inconsistencies with dosage [114], with some studies possibly not administrating a potent enough dose for cannabinoids to produce a desirable effect. Another study found no differences in spasticity relief when patients were administered placebo, Dronabinol, or whole-plant cannabis extract [112,115]. Interestingly, at the 12-month follow up, there was a significant decrease in the patients Ashworth Score, a way to measure spasticity severity and impact, in the limb given Dronabinol [115]. Another synthetic cannabinoid, Nabilone, has shown promising results at providing MS relief at low doses with patients reporting a significant decrease in pain, but no change in spasticity and/or motor functioning [22,116].

Spasticity among MS patients was greatly reduced in those who took Sativex, which is composed of 2.5% CBD and 2.7% THC by volume [117]. These promising findings have prompted researchers to further investigate the potential of cannabinoids in treating Parkinson’s and MS-related motor deficits [118]. Since 2018, the number of FDA-approved cannabinoid treatments has increased. Sativex is a cannabis-derived medication approved for use in Great Britain and Ireland for MS. Sativex, administered as an oral mucosal spray, and is a partial agonist for both cannabinoid receptors, which allows for the modulation of excitation and inhibition of various neurotransmitters, effectively providing relief to some clinical populations [119].

**Figure 4:**
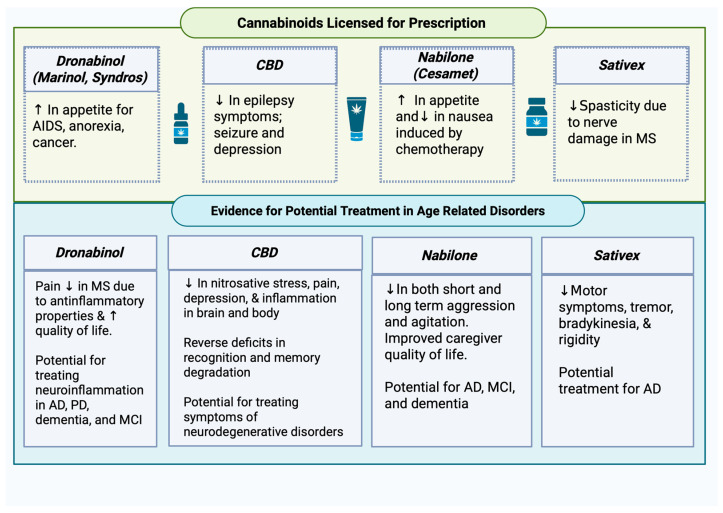
Cannabinoids licensed for prescription and evidence for potential treatment in age-related disorders. Upwards arrows indicate an increase whereas downwards arrows indicate a decrease. Created with BioRender.com.

In a study of 241 participants, Sativex was prescribed for nerve damage-induced spasticity seen in MS; approximately 80% benefited from the medication [112]. Spasticity in MS arises from damaged nerves, partially due to inflammation, and since Sativex attenuated motor symptoms of MS, it is plausible that it may also impact motor symptoms in Parkinson’s. Current cannabinoids approved for medical use related to age-related disorders can be found in Figure 4.

## 3. Overall Symptom Relief

### 3.1. Neuroprotection

Cannabinoids have been found to be neuroprotective in a variety of ways. Cells lacking CB1R are more vulnerable to damage including neuronal loss and reduced neurogenesis [120], indicating that manipulation of the ECS likely plays a role in neuronal survival, and could be used to prevent Aβ deposits and apoptosis [121,122,123]. In Van der Stelts’ study [124], injection of Aβ peptide (5 mg/kg) into the cortex resulted in inhibition of endocannabinoid reuptake via VDM-11, causing a reversal of Aβ-induced neurotoxicity and memory impairment. VDM-11 administration reversed neuronal damage via reduction in caspase-3, iNOS, COX-2, and increased calbindin [124]. This effect is seemingly dependent on early administration of the reuptake inhibitor, highlighting a temporal effect of treatment. Administration of VDM-11 later in disease progression was found to worsen memory retention—further suggesting that cannabinoid therapies are beneficial in early stages of AD. It is possible that inhibiting cannabinoid reuptake may be advantageous in terms of decreasing Aβ accumulation; however, they pose the risk of worsening progressive memory deficits seen in AD.

In the central nervous system, CB2Rs are mainly present on glial cells, as well as the brainstem, and the cerebellum [120,125]. CB2Rs are upregulated in activated microglia which is theorized to mediate the production of pro-inflammatory molecules such as interlukin-1β, ROS, and prostaglandins [126]. Evidence for this comes from the upregulation of CB2 receptors within activated microglia proximal to senile plaque deposits [92].

Bilkei-Gorzo and colleagues [127] found a decline in CB1R expression and endocannabinoid levels in aged animals, suggesting that ECS modulation could mitigate age-related symptomology. In their 2011 study, they found that 3 mg/kg of THC a day delivered via osmotic pump restored cognitive functioning, memory, and neurogenesis after 4 weeks of chronic THC administration in mature (10-month old) and old mice (18-month old) but had the opposite effect in young mice. (2-month-old). Cognitive functioning was measured using the Morris Water Maze (MWM) [128] to test spatial learning and memory retention, the Novel Object Recognition Test [129] to further assess spatial memory, and Partner Recognition Tests [130,131,132] to test social memory. THC-treated mice showed 30–50% improvement on MWM performance compared to controls. In aged mice, THC also showed restored results on the Novel Object Recognition Test to be comparable to those from young mice. Aged mice treated with THC also exhibited 40% higher social recognition compared to controls. These effects appear to be dependent on the activation of CB1R specifically, as CB1R −/− mice showed no response to THC administration. At the cellular level, THC reversed age-related synaptic loss via increased synapsin I and synaptophysin levels by 20–40%, and altered gene expressions to more closely resemble young mice—downregulating pro-aging genes (*Bdnf*) and upregulating anti-aging genes (*Casp1*)—up to 2-fold [127]. Together, these findings suggest that low-dose THC may be a viable strategy for age-related cognitive decline via CB1R activation.

### 3.2. Behavior and Cognition

Animal behavior studies also support the mediation of nNOS by the ECS. Lisboa and colleagues [133] conducted a study using iNOS knockout mice and subjected them to a Cued Fear Conditioning test (CFC). During the paradigm, mice were exposed to a neutral tone (80 dB, 2 kHz) that was paired alongside a weak electric shock of 0.5 mA for 2 s, and their freezing behavior was recorded. Rodent freezing behavior is thought to measure levels of fear or anxiety with higher incidences of freezing being indicative of an anxiety-like state [134,135,136]. iNOS knock-out (KO) mice had increased CFC scores and demonstrated increased eNOS and nNOS activity in the medial prefrontal cortex (mPFC), likely due to compensatory measures from the lack of iNOS [133]. Notably, the mPFC is primarily responsible for higher-order cognitive functions, such as memory consolidation, attention and decision-making, as well as social cognition and emotional processing, all of which are common deficits seen in clinical populations of age-related disorders [130,137,138,139]. These results support the idea that the ECS is critical to mediating affective behaviors through its interaction with the nitrergic redox system, though the effects of said interaction are dependent on the type of receptor activated and the context of its activation.

The cannabinoid agonist WIN-55 can decrease behaviors associated with anxiety at low doses via intraperitoneal (i.p.) injection, likely due to activation of CB1R [140,141]. This is possibly due to WIN-55 ability to reduce pro-inflammatory cytokines tumor necrosis factor α (TNF-α), interleukin 6 (IL-6) and interleukin 1β (IL-1β), ergo reducing the inflammatory response, and theoretically the progression of inflammation in age-related disorders like Parkinson’s and Alzheimer’s [141,142,143,144].

CBD, a CB1R negative allosteric modulator (NAM) of CB1 receptors and a CB2 receptor partial agonist [145], was found to reverse cognitive deficits in object and social recognition and memory degradation due to aging, as well as modulate chronic pain and depression-like behavior commonly seen in aging individuals or those with neurodegenerative diseases like AD, PD, and MS [109,141,146,147,148]. In a separate study by Pan and colleagues [149], CBD administration via intraperitoneal injection significantly attenuated cisplatin-induced nitrosative stress, as well as inflammation and cell death within the murine kidney, highlighting CBD’s protective role in reducing inflammation associated with aging-related diseases.

MAGL, responsible for the rapid degradation and recycling of 2-AG, has been shown to be widely beneficial in the treatment of neurodegenerative diseases, like AD, PD, and MS [143,149,150,151]. Elevated 2-AG levels due to MAGL suppression were shown to attenuate neuroinflammation, preserve synaptic proteins and hippocampal LTP, reduce amyloid-β and tau pathology, prevent neuronal loss, and ultimately improve learning and memory performance in animal models [106,107,123,150]. The inhibition of FAAH has been well studied and is shown to decrease cognitive impairment seen in Alzheimer’s disease [25,122,124].

A relatively unexplored synthetic cannabinoid, nabilone—a partial agonist for both CB1R and CB2R—reduced aggression and distress while simultaneously increasing cognitive ability over a period of six weeks in patients with moderate to severe Alzheimer’s [25]. Nabilone was effective in reducing agitation and neuropsychiatric symptoms in AD, with a medium effect size (*d* = 0.52) [25]. However, in individuals with severe cognitive deficits, nabilone worsened cognitive symptoms compared to placebo, possibly due to the disease having irreversibly damaged the brain in those with extreme cognitive deficits. Sedation is a common unwanted side effect in those using nabilone, but not significantly different than sedation experienced with other AD medications like memantine and cholinesterase inhibitors [25]. More research is needed to understand how cannabis can safely be used for patients with differing levels of cognitive deficits.

Another endocannabinoid, AEA, has been shown to relieve psychiatric symptoms such as stress-induced anxiety, and was found to inhibit panic and anxiety behaviors like fight or flight spurred by the administration of NO via FAAH inhibition in rodent models [133]. Several ongoing clinical trials are investigating CBD for mild cognitive impairment (MCI) and AD, lose-dose THC for AD, CBD as a preventative for AD, and nabilone administration for agitation in dementia and AD [152,153].

These results confirm prior studies suggesting that cannabinoid intervention can reduce inflammation and associated symptoms, improving the lives of both patients and caregivers. However, it is important to note that targeting both CB1R and CB2R may be more beneficial for controlling symptoms associated with neurodegenerative disorders, although proper dosage of both for specific conditions have not been fully parsed out yet. A summary of treatments discussed can be seen in Table 1 below.

## 4. Discussion

The effects of ECS mediation of RNS-related signaling vary substantially by the type of cannabinoid receptor activated. Cognitive and psychological symptoms can be targeted via the CB1 receptor, located abundantly in the brain. On the other hand, CB2R activation has demonstrated massive potential for the treatment of inflammation due to age-related diseases. CBD administration has been shown to improve symptoms of depression, memory degradation, and cognition—which are commonly associated with NOS-related neurodegenerative disorders [109,146,147,159,160]. Although the role of CBD in physiological symptoms such as nitrosative stress, inflammation, and cell death appears to be protective, additional studies are necessary to make further conclusions.

A critical mechanism which ECS activation may offer neuroprotection is through the direct modulation of nNOS activity and subsequent nitrosative stress. CB1R activation on presynaptic glutamatergic terminals inhibits glutamate release, in turn reducing NMDA receptor overactivation and the subsequent calcium influx, driving nNOS activity [21,22]. In a physiological environment, NMDA activation allows calcium entry, which in turn binds calmodulin and activates nNOS to produce NO at low levels appropriate for cellular signaling [39,45]. However, in neurodegenerative disorders, chronic excitotoxicity leads to sustained NMDA receptor activation, excessive calcium influx, and nNOS activation resulting in elevated NO production and the subsequent formation of ONOO- [50,52,53]. This nitrosative stress cascade contributes to protein nitration, mitochondrial dysfunction, and apoptosis [36,37]. CB1R activation may reduce nNOS overactivation indirectly via attenuating glutamatergic signaling upstream of this cascade. This is consistent with findings from Martínez-Torres and Morán [22], who demonstrated that CB1R activation provides neuroprotection via a reduction in oxidative stress in a model of glutamate excitotoxicity alongside the observation from Kim et al. that CB1R knockout mice exhibit enhanced NOS activity within the cortex [62].

While cannabinoids effects on NOS activity occurs largely through modulation of excitatory signaling, CB2R activation appears to modulate NOS through a distinct inflammation-dependent pathway. In microglia and astrocytes, pro-inflammatory signaling upregulates iNOS expression, resulting in sustained, heightened NO production that contributes to chronic nitrosative stress in neurodegenerative conditions [40,60]. CB2 agonists have been shown to suppress the inflammatory pathway and reduce iNOS expression in glial cells [63]. Additionally, AM1241, a selective CB2 agonist, attenuated microglial activation by reducing iNOS expression in rodent models [79]. Furthermore, inhibition of FAAH using PF-3845 in a TBI mouse model suppressed iNOS and COX-2 expression while simultaneously elevating AEA levels and reducing neurodegeneration [80].

Dronabinol and nabilone have been shown to improve a variety of behavioral symptoms such as anorexia, disturbed behaviors, nighttime agitation, and aggression due to neurodegeneration and age-related disorders [161,162]. However, these synthetic cannabinoids are based on the structure of THC, and can cause euphoria, paranoia, and other unwanted side effects by activation of CB1R in the brain [24].

Preclinical and clinical studies testing the effects of different cannabinoid agonists have found promising results. THCV use is not attributed to psychological effects due to its antagonism of CB1R [163] but has been found to be neuroprotective in neurodegeneration and age-related disorders [164], due to its activation of CB2 receptors. Synchronized activation of these of cannabinoid receptors within the body is possibly a more relevant area of study as medical cannabis—the most commonly prescribed form of cannabinoid treatment—is not selective with what ECS receptors it activates.

It is important to note that the effects of cannabinoid treatment are almost entirely dependent on the dose administered. In low doses, WIN-55 causes anxiolytic effects via the activation of the CB1R, whereas high doses of WIN-55 had the opposite effect, likely due to inflammation and activation of the TRPV1 receptor [165]. Interestingly, THCV may act as a CB2 agonist or antagonist depending on the dose; in high doses, THCV acts as a CB2 antagonist, whereas in low doses, THCV can act as a CB2 agonist [104]. Future studies regarding dose efficacy on a variety of cannabinoids may prove a worthy venture for researchers in treating specific symptoms/conditions. Another possible avenue of cannabinoid treatment is through the inhibition of FAAH and MAGL—the enzymes responsible for the degradation of AEA and 2-AG.

Current treatments for AD consist of cholinesterase inhibitors, NMDA receptor antagonists, and anti-amyloid therapies. Cholinesterase inhibitors such as Donepezil, Rivastigmine, and Galantamine only provide temporary cognitive improvement and are accompanied by unwanted side effects like nausea, vomiting, and bradycardia [166]. NMDA receptor agonists like Memantine have limited efficacy in later stage disease, and side effects include dizziness, confusion, and headaches. The newest anti-amyloid therapies targeting amyloid plaques show mixed efficacy, are expensive, and come with the risk of brain swelling or bleeding [167]. Although these medications are commonly prescribed, they come with serious side effects and do not stop or prevent progression of the disease. Cannabinoid treatment, on the other hand, appears to modulate early symptoms like inflammation [57], have neuroprotective properties [58], assist in general symptom relief [106], and have fewer side effects if dosed properly. Endocannabinoid treatments could also be used in conjunction with these medications to provide relief for side effects.

There are many barriers to understanding and utilizing cannabinoid-based treatments. Optimal ratios of THC:CBD, best route of administration, and longitudinal effects of treatment have yet to be investigated. This presents a need for more comprehensive studies on the interaction between the ECS and RNS to fully elucidate its mechanisms, including long-term clinical trials to assess treatment efficacy and risk factors. The promising findings of several studies demonstrate the potential of using the ECS to modulate NOS activity, particularly in the treatment of nitrosative stress-related disorders like AD and PD.

## 5. Conclusions

The interaction of the ECS and RNS systems plays a crucial role in the regulation of affect, pain, appetite, memory, and cognition and has vast therapeutic potential in treating diseases relating to NOS activity.

CB1R activation moderates glutamatergic excitotoxicity via inhibition of presynaptic glutamate release, thereby reducing calcium influx and subsequent nNOS activation [21,32,50]. Microglial CB2R activation suppresses iNOS expression, attenuating the sustained NO production that contributes to nitrosative stress [61,63]. The dual capacity of the ECS to modulate both excitotoxic (nNOS) and neuroinflammatory (iNOS) sources of NO make it a uniquely versatile target for intervention in nitrosative stress seen in neurodegenerative disorders.

Current therapies for AD and PD—such as cholinesterase inhibitors, NMDA agonists and L-DOPA—offer only symptomatic relief and are often accompanied by serious side effects such as nausea, dyskinesia, and cognitive impairment [168,169,170]. Cannabinoid treatments demonstrate multi-target potential in reducing nitrosative/oxidative stress, modulating neuroinflammation, and restoring synaptic function. Preclinical evidence from animal studies highlight the ability of cannabinoid treatments to mitigate Aβ plaque accumulation, microglial activation, and excitotoxicity, while also improving behavioral symptoms like agitation and motor dysfunction.

Many challenges remain in translating these findings into clinical use. Dose-dependent, temporal, receptor-type, and long-term effects of cannabinoid treatments have yet to be elucidated. Legal barriers such as contradictory legal status and a schedule I classification impedes the ability of researchers, consumers, and clinicians to fully understand the potential of cannabinoid treatment.

Despite these roadblocks, cannabinoid treatment offers a promising alternative to conventional treatments by addressing symptomology and the underlying molecular mechanisms of these diseases. Future research should prioritize dose and temporal-dependent effects, difference in the effects on cognition versus psychiatric symptoms, and long-term effects of cannabinoid treatment for age-related disorders. Cannabinoid treatment uniquely addresses AD and PD pathology via crosstalk between the RNS and ECS, which provides hope for disease modification as an alternative to/supplement to conventional treatments.

## Figures and Tables

**Table 1:** Cannabinoid treatment summary. Upwards arrows indicate agonists of receptors, downward arrows indicate antagonists of receptors.

Cannabinoid/RNS Agonist ↑, or Antagonist ↓	Primary Receptor Targeted	Effect ECS/RNS	Therapeutic Potential	Reference
Nabilone ↑	CB1 & CB2 ↑	Unstudied	Reduced both short- and long-term aggression in AD	[141,142,143,144]
Anandamide ↑	CB1, CB2, & TRPV1 ↑	nNOS decrease contributing to cognitive effects eNOS increase resulting in vasodilation	Vasodilation by TRPV1 activation for stroke, reduction of panic behaviors induced by NO administration	[74,154]
Dronabinol ↑	CB2 ↑	Unstudied	Pain relief for MS and appetite gain in cancer, AIDS and anorexia	[155,156,157]
2-Arachidonoylglycerol *↑*	CB1 & CB2	Nitrosative/Oxidative stress causes a compensatory increase in 2-AG, implying its role in combatting RNS/ROS, also shown to decrease neuroinflammation	2-AG administration to combat imbalance of free radicals caused by nitrosative or oxidative stress.	[150]
WIN ↑	CB1 & TRPV1	NOS implicated in itch, pain, and inflammation, possible regulation by ECS	Low doses produce anxiolytic effects, antipruritic effects, and pain relief	[141,142,143,144]
Cannabidiol (CBD) ↑	TRPV1 CB1	decrease in nitrosative stress, inflammation and cell death	Reverse deficits in object/social recognition, memory degradation, modulate chronic pain and depression-like behavior; common in progressive neurodegenerative diseases	[146,150,158]
AM281 ↓	CB1 ↓	Unstudied	Increase in CFC time, indicating ↑ in anxiety	[133]
MAGL	↑ 2-AG, MAGL inhibition decreases recycling of 2-AG	Been shown as widely beneficial in NOS-related disorders like AD and PD	MAGL suppression decrease neuroinflammation, and improve synaptic functioning in animal models	[106,107,150]
FAAH	↑ AEA due to inhibition of FAAH hydrolysis	Implicated in AD, NOS/Nitrosative stress-related disorder	Shown to decrease cognitive impairment due to AD	[122]
THCV	CB1 ↓ CB2 ↑	Implicated in PD, NOS/Nitrosative stress-related disorder	Improved motor symptoms due to PD, complete lack of unwanted psychological symptoms	[104]
THC	CB1 ↑ CB2 ↑	Implicated in ROS and NOS production	Low-dose THC restored scores of 18-month-old mice in Novel Object Recognition Task to 2-month-old levels. THC-treated old mice exhibited 40% higher social recognition. THC was found to downregulate BDNF (anti-aging) and upregulated Casp1 (pro-aging) up to two-fold.	[127]
VDM-11	CB1 ↓ CB2 ↓	Aβ role in ROS and NOS	Aβ induced neurotoxicity as well as memory impairment were reversed in rodents.	[124]
↑ - agonist	↓ - antagonist			

## Data Availability

No new data were created or analyzed in this study. Data sharing is not applicable to this article.

## Abbreviations

The following abbreviations are used in this manuscript:

ECS	endocannabinoid system
RNS	reactive nitrosative species
AD	Alzheimer’s disease
PD	Parkinson’s disease
GPCR	g protein-coupled receptor
FAAH	fatty acid amide hydrolase
AEA	anandamide
2-AG	2-arachidonoylglycerol
THC	tetrahydrocannabinol
CBD	Cannabidiol
NAPE	N-acyl-phosphatidylethanolamine
NAPE-PLD	N-acyl-phosphatidylethanolamine-hydrolyzing phospholipase D
PLC	phospholipase C
DAG	diacylglycerol
cAMP	cyclic adenosine monophosphate
PKA	Protein kinase A
CAMKII	calcium/calmodulin-dependent protein kinase II
MAPK	Mitogen activated protein kinase
CNS	central nervous system
EPM	elevated plus maze
ATP	Adenosine triphosphate
mtNOS	mitochondrial nitric oxide synthase
RNS	Reactive nitrogen species
ROS	reactive oxygen species
AO	antioxidants
MS	multiple sclerosis
RyR’s	Ryanodine receptors
ER	Endoplasmic recticulum
MERC’s	mitochondria endoplasmic reticulum contact sites
MCU	mitochondrial calcium uniporter
mPTP	mitochondrial permeability transition pore
NO	nitric oxide
NOS	nitric oxide synthase
nNOS	neuronal nitric oxide synthase
eNOS	endothelial nitric oxide synthase
iNOS	inducible nitric oxide synthase
LTP	long-term potentiation
NMDA	N-methyl-D-aspartate
cGMP	Cyclic guanosine monophosphate
L-NAME	N-omega-nitro-L-arginine methyl ester
fEPSP’s	field excitatory post-synaptic potentials
PKC	protein kinase C
PKG	protein kinase G
ONOO-	superoxide anion form peroxynitrite
MAGL	monoacylglycerol lipase
OA	osteoarthritis
THCV	tetrahydrocannabivarin
MWM	Morris Water Maze
CFC	Cued fear conditioning
KO	knock out
mPFC	medial prefrontal cortex
i.p.	Intraperitoneal
TNF-α	tumor necrosis factor alpha
IL-6	interleukin 6
IL-1β	interleukin 1-β
NAM	Negative allosteric modulator
MCI	mild cognitive impairment
